# Comprehension as Purification in Reading

**DOI:** 10.3390/e27121261

**Published:** 2025-12-17

**Authors:** Miho Fuyama

**Affiliations:** 1 College of Letters, Ritsumeikan University, 56-1 Toujiin Kitamachi Kita-ku, Kyoto 603-8577, Japan; miho02@sj9.so-net.ne.jp; Tel.: +81-075-465-8187; 2 School of Information Systems, Queensland University of Technology, Brisbane 4000, Australia

**Keywords:** comprehension, measurement, purification, quantum cognition, reading, von Neumann entropy

## Abstract

When reading a novel or poem, readers sometimes gain comprehension or experiences that cannot be expressed in language yet are felt as holistic. Previous studies focused on the linguistically expressible aspects of text comprehension. In this study, we propose a new hypothesis, the *purification comprehension hypothesis*, that seeks to explain how a reader constructs indescribable and coherent comprehension using quantum probability theory. This hypothesis regards the reading process as purification, in which the reader’s initial interpretation state is mixed, and the reader incorporates external systems, such as the interpretation of other parts of the text or prior knowledge, to purify their state. Therefore, the dimensionality of the state increases and von Neumann entropy decreases through purification. We also highlight two types of reading based on this hypothesis: purification and deterministic. Our model contributes to studies on reading by bridging humanities and scientific studies, provides implications for cognition models that aim to minimize Shannon entropy, and has the potential to apply cognition related to other modalities and media, such as music and art.

## 1. Comprehension Beyond Words Evoked by Words

Readers can imagine fruitful images and construct abstract comprehension that is sometimes inexpressible, indescribable, or ineffable. “Quoth the Raven ‘Nevermore.’ ” from Poe’s famous poem *The Raven* [1] can evoke diverse and inexpressible interpretations. Their interpretation can be polysemous and inexpressible but not random. We have specific comprehension according to the text and our knowledge that is indescribable by words. (In this article, we basically use interpretation for local passages or scenes, and comprehension for the integrated understanding of the entire text. However, this distinction is not applied strictly. When referring to previous studies, we follow the terminology used in those works.)

These indeterminate interpretations for each word, sentence, and chapter or each event, character, and atmosphere are combined to construct holistic comprehension of a text in a coherent manner. Gadamer [2] proposed the concept of the *hermeneutic circle*, which is the hierarchical and cyclical structure of understanding between the parts and whole of a text. The hermeneutic circle suggests that any interpretation of a part of the text cannot be determined in isolation but depends on other parts as well as higher- and lower- level structures of interpretation.

Regarding the construction of indescribable and coherent comprehension, Iser [3] also discussed the *gap*, which is the unwritten part or implication of the text. These gaps involve readers in the interpretation process and stimulate their creativity. The text contains numerous gaps; some can be inferred by the reader, whereas others may remain indeterminate blanks. Iser [3] argued that there is no correct interpretation for these gaps, nor is there a single “correct” interpretation for the text as a whole. He also emphasized that reading is a process of constructing a coherent interpretation that necessarily includes gaps, regardless of whether they are inferred or left open. The indeterminate interpretation related to the gaps emerges as an indescribable interpretation, and the construction of coherent interpretation corresponds to constructing holistic comprehension. Eco [4] proposed a related concept *Openness*, which refers to ambiguous or indeterminate elements or aspects of modern art or texts that involve the viewer or reader in its appreciation.

Contrary to intensive discussions in the humanities, this aspect of reading, which is constructing indescribable and coherent comprehension, has not been sufficiently studied in psychology and cognitive science. (This study focuses on indescribable comprehension evoked by text. More broadly, indescribable mental representations have long been discussed in psychoanalysis and cognitive science. In the field of quantum cognition, related discussions have also appeared, for example, in studies such as Battilotti et al. [5] and Bruza [6].) In psychology and cognitive science studies, it has been suggested that readers have three levels of comprehension; surface level, text level, and situation model level [7,8]. The situation model includes all the inferences that go beyond the concepts explicitly mentioned in the text [8] and the relevant level of the discussion.

Although the situation model level, where readers use their knowledge to construct coherent comprehension, corresponds to constructing interpretation and imagination, previous studies assumed that the representation of this level can be written in language. For example, *the event indexing model* [9], one of the most influential models of text comprehension [10,11], hypothesizes that readers construct their understanding based on six (or five) dimensions: cause, character, goal, object, space, and time. Based on their hypothesis, readers track the value of these dimensions and, roughly speaking, this is the comprehension process. For instance, in Zacks et al. [11], the sentence “[Mrs. Birch] sent through the front door into the kitchen” represents content about the cause and space; readers regard the “kitchen” as the value of the space. Empirical studies support this hypothesis. For example, when large changes occur among these dimensions, the reading time increases, and readers presume that this is the endpoint of the event [11]. These results support the idea that readers use the information on these dimensions to segment an event.

In terms of coherence, numerous psychological and cognitive studies on reading assumed that “coherence” is significant for the understanding of the entire text. Van Dijk et al. [7] proposed the concept of *Macrostructure*, which is defined as a product of inference characterized by reducing the text information to its essential message. This process follows three rules: deletion, generalization, and construction. These rules convert text into propositions and make them more concise propositional representations. Therefore, they assumed that readers’ comprehension could be represented as a proposition and implicitly as natural language.

However, in terms of the event-indexing model, which dimension is associated with “Nevermore?” In the poem, “Nevermore” is obviously a critical word. The word “Nevermore” appears several times in the poem. If the interpretation of each part is indescribable, it cannot reduce the proposition, especially using natural language representation; thus, the macrostructure cannot be constructed using the above method.

This is not limited to the case of poetry but also extends to ordinary stories. Miall and Kuiken [12] pointed out that stylistic features known as foregrounding, such as metaphors, capitalization, and consonance, increase reading time and evoke readers’ feelings. These features cannot be accommodated within any dimension of the event-indexing model and are omitted in the construction of the macrostructure following the above three rules. However, the results by Miall and Kuiken [12] suggested that such stylistic features play a significant role in text comprehension, and it appears difficult to explain the meanings of these features using language.

Although the situation, event-indexing, and macrostructure featured models may be valid from certain perspectives, they do not fully account for indescribable comprehension and their coherent holistic structure. This aspect, which is related to the reading theory proposed by Iser [3], Eco [4], and foregrounding that prompts defamiliarization require another type of model that can explain how readers construct holistic comprehension, which sometimes goes beyond the words themselves.

This study proposes a “purification comprehension hypothesis.” This hypothesis presents a potential model of how readers construct an indescribable and holistic comprehension of texts. First, we provide a brief outline of the overall hypothesis for a clear perspective. We model the reader’s comprehension state and process using the quantum probability theory, a more general probability theory that includes the classical probability theory, which is the standard probability theory traditionally used in modeling cognition. Our main hypothesis is that the reading process can be modeled as the purification of the reader’s state. Based on the quantum probability theory, there are only two types of states: pure and mixed. The text presents information corresponding to the mixed state. Readers purify this mixed state by adding an external system that corresponds with other parts of the text or with their knowledge. Because it is difficult or impossible to interpret nearly all pure states using our natural language because of their indeterminacy, readers’ interpretations, understandings, and experiences can go beyond words. This purified system can be regarded as holistic with respect to certain quantum types of correlations between the subsystems.

In Section 2, we explain the mathematical concepts necessary for the proposed model with an example of its application in quantum cognition and simple examples from quantum information processing. This section is presented as the background. Readers familiar with the fundamentals of quantum theory and quantum information processing may choose to disregard it. In Section 3, we propose a more detailed purification comprehension hypothesis and its implications for related areas. Section 3.4 presents the connection to other cognitive models that assume that the cognitive system aims to reduce uncertainty or predictive errors. Finally, we discuss future work to improve and test our hypothesis and the contributions of the study (Section 4).

## 2. Brief Explanation of Mathematical Concept with Its Application on Quantum Cognition

We employ quantum probability theory (since quantum probability theory includes the classical probability theory, all the models written by classical ones can be translated into quantum ones) to model the comprehension state but do not assume that our brain or body has a quantum physical state. This position align with the “quantum cognition [13]” or “quantum-like cognition [14].” This article briefly introduces the mathematical concepts necessary to propose our hypotheses. For a more detailed explanation and discussion of quantum(–like) cognition, please refer to Busemeyer and Bruza [13], Khrennikov [14], Yearsley [15]. Further quantum cognitive studies of text comprehension relevant to our hypothesis are introduced in Section 4 to discuss its verifiability.

### 2.1. Pure State and Mixed State, Observable, and Measurement

There are two types of states based on the quantum probability theory. One is a pure state and the other is a mixed state. Mathematically, a state is defined as a function of *-algebra *A* that satisfies linearity, positivity, and the condition of returning 1 to the unit element. A pair consisting of this state and the *-algebra *A*, yields the algebraic probability theory, also known as the quantum probability theory. In this paper, we do not pursue a formal mathematical definition but rather proceed based on the following implementation that is frequently employed in quantum(–like) cognition.

As in the studies on quantum cognition, states implement a state vector in the Hilbert space [13]. In this implementation, the state remains a function that returns the probability distribution for each observable. For example, if we ask for the interpretation of a sentence, the observable is the interpretation (defining what the observable is in the cognition is a profound question, but we do not address it in this paper). The reader’s interpretation state returns their interpretation stochastically for the observable. The main difference between the quantum and classical probability theories is whether a joint probability distribution can be assumed for all pairs of observables. In classical probability theory, this assumption always holds. However, in quantum probability theory, if the observables do not commute with each other, no joint probability distribution can be defined.

In this implementation, the pure state is defined as the all state, which can be represented as the composition of the pure state. The mixed state was defined as the other states.

For example, let us consider a pure state $|\psi_{p}\rangle$ that can be expressed as a composition of two-level states |0〉, |1〉. This constitutes a superposition of the two states.

(1) $$\begin{array}{c} |\psi_{p}\rangle =\alpha |0\rangle +\beta |1\rangle \;\alpha ,\beta \in {C,\;|\alpha |}^{2}+{\left|\beta \right|}^{2}=1 \end{array}$$

α and β are not the probability itself but the probability amplitude. For instance, the two basis states may correspond to spin-up and spin-down in physics, and to literal and metaphorical interpretations in the case of figurative sentences [16].

When we measure the observable *A*, the expectation value is

(2) $$\begin{array}{c} \langle \psi_{p}|A|\psi_{p}\rangle ={\left|\alpha \right|}^{2}\langle 0|A|0\rangle +{\left|\beta \right|}^{2}\langle 1|A|1\rangle +\alpha^{*}\beta \langle 0|A|1\rangle +\beta^{*}\alpha \langle 1|A|0\rangle \end{array}$$

The first two terms correspond to the probability distributions obtained when measuring the observable *A* in states |0〉 and |1〉. The latter two terms are interference terms, which indicate that if the vectors |0〉 and |1〉 are not orthogonal, the measurement outcomes for the superposed state deviate from the probabilistic weighted sum of the outputs of the individual states (the first two terms).

The first two terms correspond to the classical case; that is, the weighted sum of each state distribution.

(3) $$\begin{array}{c} \langle \psi_{m}|A|\psi_{m}\rangle ={\left|\alpha \right|}^{2}\langle 0|A|0\rangle +{\left|\beta \right|}^{2}\langle 1|A|1\rangle \end{array}$$

Can we express the state vector $|\psi_{m}\rangle$? If the vectors |0〉 and |1〉 are not orthogonal, the state cannot be expressed as a state vector. This state is classified as a mixed state, and a density matrix is required to represent it uniquely. The density matrix is represented as

(4) $$\begin{array}{c} \rho =\Sigma_{i}p_{i}|\psi_{i}\rangle \langle \psi_{i}| \end{array}$$

$p_{i}$ represents the probability that the state is $|\psi_{i}\rangle$. If the state is pure, the density matrix can be represented as ρ=|ψ〉〈ψ|.

The pure state Equation (2) can be expressed using the density matrix as follows:

(5) $$\begin{array}{c} \begin{array}{cc} \rho_{p} & =|\psi_{p}\rangle \langle \psi_{p}|\\ \; & ={\left|\alpha \right|}^{2}|0\rangle \langle 0|+\alpha \beta^{*}|0\rangle \langle 1|+\alpha^{*}\beta |1\rangle \langle 0|+{\left|\beta \right|}^{2}|1\rangle \langle 1|\\ \; & =\left[\begin{array}{cc} {\left|\alpha \right|}^{2} & \alpha \beta^{*}\\ \alpha^{*}\beta & {\left|\beta \right|}^{2}\; \end{array}\right] \end{array} \end{array}$$

The mixed state Equation (3) can be expressed using the density matrix as follows:

(6) $$\begin{array}{c} \begin{array}{cc} \rho_{m} & =|\psi_{m}\rangle \langle \psi_{m}|\\ \; & ={\left|\alpha \right|}^{2}|0\rangle \langle 0|+{\left|\beta \right|}^{2}|1\rangle \langle 1|\\ \; & =\left[\begin{array}{cc} {\left|\alpha \right|}^{2} & 0\\ 0 & {\left|\beta \right|}^{2}\; \end{array}\right] \end{array} \end{array}$$

Equation (3) may appear to allow the interpretation that the mixed state corresponds to a preparation in which pure states |0〉 and |1〉 are generated with proportions $\alpha^{2}$ and $\beta^{2}$, respectively. This procedure can produce the mixed state. However, there are infinitely numerous ways to construct the same mixed state if one identifies the states that yield the same probability distribution. For example, if we prepare a pure state $|\pm \rangle :=\frac{1}{\sqrt{2}}(|0\rangle \pm |1\rangle$ with equal probability 1/2, the resulting mixed state is identical.

### 2.2. Composite System

The reader integrates interpretations of individual words, sentences, paragraphs, and so on to construct the entire comprehension of a story. We employed a composite system to represent the entire comprehension state (The hierarchical structure cannot be represented through this model. We will discuss this point in the Section 4).

Let us consider a pure state ψ of two d-level systems, A and B. Here, *U* and *V* are the local unitary transformations. Based on the Schmidt decomposition, ψ can be written as follows.

(7) $$\begin{array}{c} \left(U⊗V\right)|\psi \rangle =\Sigma_{i=0}^{d-1}\sqrt{p_{i}}{|i\rangle }_{A}{|i\rangle }_{B} \end{array}$$

where $p_{i}>0$ for i=0,…,d−1 and $\Sigma_{i=0}^{d-1}p_{i}=1$. If one of the Schmidt coefficients, say $p_{k}$, equals 1 while all the other $p_{i}\left(i\neq k\right)$ are zero, then the state can be expressed as a product of the states of the two subsystems, referred to as the product state. Otherwise, the state cannot be expressed as a product and becomes an entangled state. In this case, quantum correlations exist between the two subsystems.

Let us now consider the *Bell State* as an example, which consists of entangled states of two-level (|0〉 and |1〉) systems. One of the Bell states is:

(8) $$\begin{array}{c} |\psi^{+}\rangle =\frac{1}{\sqrt{2}}\left({|0\rangle }_{A}{|0\rangle }_{B}+{|1\rangle }_{A}{|1\rangle }_{B}\right) \end{array}$$

The corresponding density matrix is

(9) $$\begin{array}{c} \rho \left(\psi^{+}\right)=\frac{1}{2}\left[\begin{array}{cccc} 1 & 0 & 0 & 1\\ 0 & 0 & 0 & 0\\ 0 & 0 & 0 & 0\\ 1 & 0 & 0 & 1 \end{array}\right] \end{array}$$

In this Bell state, because of the quantum correlation between states A and B, if state A is measured as |0〉 (|1〉), then state B is also measured as |0〉 (|1〉).

In quantum(–like) cognition, numerous studies have used entanglement representations to model cognition, such as decision-making, language understanding, and conceptual understanding [17,18]. For example, Bruza et al. [18] modeled conceptual combinations using an entanglement representation. They focused on polysemous word pairs such as “BAT BOXER.” They assumed that if participants understood the word “BAT” in the context of sports (animal), then they would also be likely to understand the word “BOXER” in the same context. Therefore, such a conceptual combination can be modeled as an entanglement between the two concepts. In this example, modeling with entanglement is a candidate for representing structures with basis-independent correlations.

### 2.3. Induced Decoherence and Purification of the State

When an entangled state is measured or when noise enters the system, coherence can be reduced and the entanglement state can be partially degraded, possibly approaching a mixed state with only classical correlations.

We observe the Density matrix after performing a partial measurement on system *B* in the Bell state $\rho (\psi^{+})$. When observing system *B*, it suffices to take a partial trace of system *B*, as follows:

(10) $$\begin{array}{c} Tr_{B}\left(\rho \left(\psi^{+}\right)\right)=\frac{1}{2}\left[\begin{array}{cc} 1 & 0\\ 0 & 1 \end{array}\right] \end{array}$$

This mixed state is equal to $\rho =\frac{1}{2}\left(|1\rangle \langle 1|+|0\rangle \langle 0|\right)$.

In contrast with this process, mathematically, any mixed state can be purified into a pure state by adding an appropriate external system. Let us consider $\rho_{S}$ as an arbitrary density matrix in system *S* and system *A* with a Hilbert space $H_{A}$ whose dimension *d* is the same as that of $H_{S}$. The spectral decomposition of $\rho_{S}$ is represented by $\Sigma_{i=1}^{d}q_{i}|s_{i}\rangle \langle s_{i}|$, and ${|a_{i}\rangle }_{i=1}^{d}$ is set as the appropriate orthonormal system basis. Because $|\psi \rangle :=\Sigma_{i}\sqrt{q_{i}}|s_{i}\rangle ⊗|a_{i}\rangle$ is a unit vector, $\rho_{SA}=|\psi \rangle \langle \psi |$ is a pure state on $H_{SA}$. Then $Tr_{A}\rho_{SA}=\Sigma_{ij}\sqrt{q_{i}}\sqrt{q_{j}}\left(Tr_{A}|a_{i}\rangle \langle a_{j}|\right)|s_{i}\rangle \langle s_{j}|=\Sigma_{i}q_{i}|s_{i}\rangle \langle s_{i}|$ using $Tr_{A}|a_{i}\rangle \langle a_{j}|=\langle a_{j}|a_{i}\rangle =\delta_{ij}$. Therefore, $\rho_{S}$ is the reduced density matrix of $\rho_{SA}$ (see Ishizaka et al. [19]). This method is one possible way of purifying a given mixed state, and there exist infinite other ways to purify it. It should be noted that this method leads to a product state, and thus does not result in an entangled state.

### 2.4. Purity

Is there a measure that characterizes how pure the state is? A commonly used measure is *purity*. Given a density matrix ρ in Hilbert space *H*, purity is defined as $Tr(\rho^{2})$. This satisfies the following inequality where *d* denotes the dimensions of the Hilbert space:

(11) $$\begin{array}{c} \frac{1}{d}\leq Tr\left(\rho^{2}\right)\leq 1 \end{array}$$

The equality $Tr(\rho^{2})=1$ holds if and only if the state is pure.

## 3. Purification Hypothesis

### 3.1. Modeling the Interpretation with Indeterminacy and Indescribability

The purification hypothesis assumes that the two types of indeterminacy are related to interpretations beyond words. These assumptions are the basis of the purification hypothesis but not the original assumption. Fuyama [16,20] modeled the metaphor and story comprehension based on this indeterminacy. Bruza and Woods [21] focused on an interpretation made at a sublinguistic level in communication contexts, and modeled interpretation as a collapse of superposition states of word meaning.

When the quantum probability theory is employed to model cognition, two types of indeterminacy arise. First, we cannot assume a joint probability among every observable at once, owing to the non-commutativity of the observables. Therefore, it is impossible to assume that every aspect of our mind corresponding to each observables, such as the interpretation of the character, scene, and theme, was set before the measurement. Second, even when we decide on a fixed observable to measure, the results of the measurement are stochastically distributed. That is, a state (mind state) is a function that returns a probability distribution rather than a decisive value. The interference terms in the superposition state are clear examples of these two types of indeterminacy. The interference terms arise from the noncommutative observable, and the measurement output is distributed.

Regarding indescribable interpretations, we believe that these indeterminacies cause comprehension beyond words. Because of the first type, assuming that a text can be interpreted from multiple perspectives that do not necessarily commute to each other, the reader’s interpretation inevitably includes indeterminacy.

The second type (and first type) of indeterminacy is related to the relationship between the reader’s interpretation state and the language semantic space, which corresponds to the basis of observables. In most cases, they were not aligned or were orthogonal. Furthermore, in the semantic space, representations of meanings usually do not appear orthogonal to each other, considering several semantic models [22]. Therefore, when readers want to express their interpretation of a story, they must combine many words that are noncommutative. Their expression as a distribution contains interference terms, but a single measurement cannot express their states. In other words, the pure-mind state cannot be reduced to a weighted sum of classical states.

### 3.2. Reading Process as a Purification

At this point, we propose the purification comprehension hypothesis. In this hypothesis, we use the quantum probability theory to represent the reader’s comprehension state as a multipartite state in a composite system that contains a partial state corresponding to each interpretation for different parts or targets of the story (these interpretations and parts of the texts may not represent the natural language). When reading a book, the reader’s interpretation state is mixed because they have not found a relationship with each partial interpretation; they can understand each sentence, each event, or characters’ feelings, but they have not determined the entire coherent structure of the story. We hypothesized that the reading process could be regarded as a purification of the interpretation state. The readers explore the coherent structure based on their knowledge of the real world, reading experiences, and meta-messages embedded in the text. This process corresponds with the exploration of an appropriate external system and its relationships to make the entire state pure, that is, purification. According to Iser [3], Eco [4], any text has indeterminacy, which is the space for interpretation that enhances readers’ immersive and aesthetic experiences. These indeterminacies, or interpretive spaces, correspond to the spaces that are complemented by external systems. Purity is one of the indices of this purification comprehension process (von Neumann entropy is also an index; see Section 3.4).

Based on this model, readers cannot express their comprehension state using natural language. There are three reasons for this finding. First, they are usually in superposed states and our limited words, which become the basis of the measurement, rarely align with this state vector. In this case, the interference term cannot be precisely represented. Second, even if readers can represent their interpretation for one event or sentence, their whole interpretation cannot be expressed simultaneously because of the noncommutativity of the observables; we cannot consider the joint probability distribution for all the observables (This leads the order effects that mean the expressions or reports about the interpretations are changed according to the order of expression [20]).

We assume that reporting the readers’ state should be a measurement because they have to measure themselves and output their states. In this case, reporting(expressing) causes decoherence in the state, which becomes a mixed state. Once readers express themselves or interact with others or the outside environment, their pure interpretation states are broken down into a mixed state. This situation appears to align with the view that readers’ immersion states can be disrupted by others calling out, and that at times, they may prefer to retain their experiences or understanding of themselves rather than reporting them. (Based on the quantum probability theory, any measurement alters the state.).

### 3.3. Example

Let us consider, in its most simplified form, a reading consisting of interpretations of the two events. This example corresponds to the Bell state introduced in Section 2.

For this example, we employed the novel named “Manazuru” written by Hiromi Kawakami who is a Japanese novelist. In the climax, the late husband of the protagonist asks her whether she will “come,” and the protagonist replies “ikitai.” In Japanese, this utterance has two possible interpretations: one is ikitai in the sense of “I want to go,” which here implies a wish to join the dead (thus, to die); the other is ikitai in the sense of “I want to live,” expressing her desire to remain alive. The protagonist tells her husband that she herself does not know the meaning she intended, and the scene passes without her being able to decide.

In this scene, the protagonist’s attitude toward life or death can be represented as state $|\psi_{A}\rangle$, whereas her attitude toward either her husband or family can be represented as another state, $|\psi_{B}\rangle$. The composite system consisting of these subsystems is denoted as $|\psi_{AB}\rangle$.

Let $|\psi_{A}\rangle$ be defined such that |0〉 represents “wanting to go (wanting to die)” and |1〉 represents “wanting to live.” Similarly, let $|\psi_{B}\rangle$ be defined such that |0〉 represents “wanting to be with her husband” and |1〉 represents “wanting to be with her present family.”

From the overall structure of the narrative, a coherent interpretation is that if she dies, she will be with her husband, whereas if she lives, she will remain with her family. Accordingly, the possible global interpretive state vector is $|\psi_{AB}\rangle =\alpha |00\rangle +\beta |11\rangle$ (α, $\beta \;\in {\;C,\;|\alpha |}^{2}+{\left|\beta \right|}^{2}=1$).

In this setting, each state, $|\psi_{A}\rangle$, $|\psi_{B}\rangle$, and also $|\psi_{AB}\rangle$ is a superposition and a pure state (for example, $|\psi_{A}\rangle =\alpha_{a}|0\rangle +\beta_{a}|1\rangle$, $\alpha_{a}$, $\beta_{a}\in C,\;|\alpha_{a}{|}^{2}+{\left|\beta_{b}\right|}^{2}=1$). However, what happens if one reads only this sentence without understanding the overall structure of the narrative? In this “real” world, the woman is either dead or alive; she is not in a superposition but rather in a probabilistic mixture, represented as a mixed state. The corresponding density matrix is expressed as $\rho_{A}={\left|\alpha \right|}^{2}|0\rangle \langle 0|+\beta^{2}|1\rangle \langle 1|$.

According to the purification comprehension hypothesis, reading is conceived as the process of constructing a pure state from a mixed state (or just a classical probabilistic mixture state) by identifying the relational structure between other partial interpretations (in this example, whether she wishes to be with her husband or her present family). From this perspective, comprehension involves the construction of a pure state $|\psi_{AB}\rangle =\alpha |00\rangle +\beta |11\rangle$.

In the density matrix form, the mixed state is expressed as

(12) $$\rho_{A}=\left[\begin{array}{cc} {\left|\alpha \right|}^{2} & 0\\ 0 & {\left|\beta \right|}^{2}\; \end{array}\right]$$

whereas the purified matrix, which reflects the overall relational structure, is given by:

(13) $$\rho_{AB}=\left[\begin{array}{cccc} {\left|\alpha \right|}^{2} & 0 & 0 & \alpha \beta^{*}\\ 0 & 0 & 0 & 0\\ 0 & 0 & 0 & 0\\ \alpha^{*}\beta & 0 & 0 & {\left|\beta \right|}^{2}\; \end{array}\right]$$

In this case, identifying the correlation expressed in off-diagonal elements constitutes a possible purification process. In the present example, the content corresponding to $|\psi_{B}\rangle$ is described to some extent in the novel; however, there may also be cases in which this is not explicitly presented. Furthermore, while this example provides a verbal explanation of the interpretation and meaning of the state vector, it is not always possible to express either global or subsystem interpretations and their state vectors linguistically; in many cases, this is impossible.

It may not seem too difficult to find the correlation and construct $\rho_{AB}$ from $\rho_{A}$. However, one mixed state is achieved using several combinations of pure states. This creates a difficulty that corresponds with estimating the density matrix of a mixed state (See Section 2.1). Readers must also find an appropriate external system that corresponds to other sentence interpretations or to their knowledge. If we regard the interpretation of a single event/phenomenon as a pure state, to purify the mixed state, readers try to pick up these interpretations and find the appropriate relationships. However, during reading, there are a lot of events and their combinations. Furthermore, it is undecided on how to draw the boundary of a single object of interpretation; for example, what exactly constitutes one event.

This difficulty aligns with our intuition of the comprehension process. When we read a novel, we do not know which scenes, events, utterances, descriptions, or words are important for understanding it. As we read, we consider the things that are significant as well as the contextual relations among them.

In other words, although mathematically, any external system whose dimensions are the same as those of the original system can be used for purification, the reading comprehension process should be more restricted. This study did not specify such constraints. However, further work is needed to investigate the conditions that purification must satisfy in the context of reading comprehension.

### 3.4. Von Neumann Entropy and Shannon Entropy During Purification

Currently, an increasing number of cognition models are based on the assumption that humans want to reduce the entropy inside models or simply in the brain or body, such as the *free energy principle* [23]. The purification comprehension model can be related to these models through von Neumann entropy.

Based on the quantum probability theory, von Neumann entropy is an index of the amount of information or complexity of a state corresponding to the Shannon entropy. von Neumann entropy is defined as follows:

(14) $$\begin{array}{c} S=-Tr(\rho \ln(\rho )) \end{array}$$

ρ is the density matrix of state. von Neumann entropy is a measure of statistical uncertainty in a quantum system and can be interpreted as a measure of complexity, reflecting the extent of a probabilistic mixture. When the state is pure, the von Neumann entropy is zero. Using the Taylor expansion [24],

(15) $$\begin{array}{cc} S & =Tr(\rho -\rho^{2})+\mathrm{higher}\;\mathrm{order}\;\mathrm{terms}\\ \; & =1-\gamma +\mathrm{higher}\;\mathrm{order}\;\mathrm{terms} \end{array}$$

Note that γ denotes purity.

Based on the purification comprehension hypothesis, we hypothesized that readers purify their interpretive state, corresponding to an increase in purity toward one. By definition, von Neumann entropy takes the value of zero for pure states, and from Equation (15), it follows that increasing the purity toward one corresponds to decreasing the von Neumann entropy toward zero.

Purity and von Neumann entropy are both basis-independent, and are features of the state itself. Contrary to von Neumann entropy, when we define Shannon entropy relative to the mind state, because it is generally an index of one probability distribution corresponding to one random variable; we need corresponding observables, not only the reader’s mind state. If we employ such a definition, the Shannon entropy of the state and the observable should be basis-dependent. Therefore, even if the reader’s interpretation state is pure and the von Neumann entropy is zero, the Shannon entropy corresponding to some observables cannot be zero.

More precisely, Brody and Hughston [25] defined the Shannon entropy of a system as the uncertainty of the observable $\hat{X}$ in the state, in accordance with the definition discussed above. They demonstrated that inducing decoherence reduces the Shannon entropy for any observable, while increasing von Neumann entropy.

This difference between von Neumann and Shannon entropies results in different predictions of the models employing each entropy as an index to reduce. This difference reflects the core of cognitive modeling. One of the difficulties of cognitive models that minimize Shannon entropy lies in the arbitrariness of choosing the random variable in Shannon entropy that should be minimized (in fact, the core of such models). This difficulty corresponds to the question of the observables that should be selected. In contrast, when von Neumann entropy is employed as the index, arbitrariness lies in the definition of the system and what we regard as the state. This is the core of our model, which the reader attempts to purify. However, it should be noted that this arbitrariness is also present in models based on Shannon entropy.

How can we connect the purification-type models and those employing Shannon entropy? Brody and Hughston [25] argued that when any pure state is taken as the reference, decoherence (including measurement) decreases the Shannon entropy while increasing the von Neumann entropy, regardless of the observable considered. It may seem difficult to determine the connection between the two types of models; however, because purification can be realized in various ways, depending on the kind of external systems that are introduced and the extent to which the state can be purified, we argue that the two types of models can be reconciled without contradiction. If, starting from a given mixed state, one considers multiple possible pure states obtained through purification, together with various sets of observables, then it may turn out that Shannon entropy is reduced relative to the measurement of the original mixed state; there may exist mixed states in which the measurement yields even lower Shannon entropy from multiple possible pure states.

Based on these distinctions, von Neumann entropy can be regarded as reflecting the coherence of the information structure of the state itself, or its capacity to generate information, with a lower von Neumann entropy corresponding to a higher degree of these properties. In contrast, Shannon entropy reflects the amount of information realizable with respect to a given observable. However, as Brody and Hughston [25] suggested a somewhat different interpretation, further discussion on the interpretation of both entropies is required in the context of our models.

### 3.5. Deterministic Reading as a Measurement Process

We propose purification as a reading comprehension process. However, is this the only way to read text? We assume that at least one more process, that is measurement, is possible. This is the opposite of the purification process, because measurement causes decoherence and breaks the pure state.

Maryanne [26] discussed two types of reading. One is complex and deep reading, such as *Das Glasperlenspiel* [27], and the other is quick and shallow reading, such as going through numerous e-mails or short messages. Maryanne [26] suggested that once we engage only quick and shallow reading, we will lose the ability to read deeply and complexly.

We assumed that complex and deep readings are related to purification, and quick and shallow readings are related to measurement processes. In contrast to reading novels, the characteristic of the latter type of reading is that we have to decide on one interpretation and meaning of the text (and react to it as soon as possible). These deterministic readings are also found in several other situations. For example, when reading textbooks or how-to books, we sometimes find summaries that help us understand the aim of a chapter or section. These summaries confirm our interpretation.

Because decision-making is regarded as a measurement [13] assumed that deciding one or more interpretations as the correct or appropriate understanding of a text could be regarded as a measurement. Based on this assumption, during deterministic reading, readers frequently measure their own interpretative state, which induces decoherence and loss of purity, and when viewed from the perspective of von Neumann entropy, a loss of information. However, this type of reading is rational considering the cognitive load or immediate communication situation.

Deep and complex reading requires the ability to maintain indeterminacy among multiple interpretations and to purify one’s interpretation state, including relevant text information, in a coherent manner. In this type of reading, we must construct complete comprehension without determining the partial interpretation of each included system.

Real reading could be any of these two types. Even highly skilled readers are unlikely to read without creating any interpretations at all. Readers must measure irrelevant aspects to conserve their cognitive resources while preserving essential indeterminacy for overall interpretation.

## 4. Discussion

In this study, we propose the purification comprehension hypothesis as a new model of text comprehension. Whereas the previous sections primarily focused on the theoretical framework, this section discusses future directions, including possible methods for empirical verification and algorithmic implementation.

To illustrate the study’s potential verifiability, we introduce empirical studies related to this hypothesis in Section 4.1. For a more comprehensive review of quantum cognitive research on language comprehension, we refer readers to Chapter 11 of Busemeyer and Bruza [13].

### 4.1. Approach to Test the Hypothesis

To test our hypothesis, the most direct approach would be to estimate the reader’s comprehension state. However, estimating purity requires the reconstruction of a density matrix from the measurement outcomes, and no robust method currently exists for the mental state (if it is reasonable to regard the comprehension state of the participants as distributed normally, and assume the average comprehension state, we could estimate the density matrix using quantum state statistical inference or the tomography technique). Although an entanglement measure is also a possible index because it reflects the amount of quantum-type correlation, this measure is difficult to estimate directly.

Therefore, as a first step toward verifying our hypothesis, we suggested testing whether the state is superposed and whether it is entangled at some points during reading.

Regarding the superposition state, the order effect and qq-equality are usually employed as indices for testing [16,28]. However, Ozawa and Khrennikov [29] and Busemeyer et al. [30] suggested that there are other types of models that do not require the superposition state but can explain the order effect and qq-equality. Although these studies indicated that the order effect and qq-equality are not sufficient conditions to test the existence of a superposed state, we assumed that these indices still provide supportive (but not sufficient) evidence for the existence of a superposition state.

Fuyama [20] conducted an experiment regarding text comprehension aligned with this method, and the significant order effect was measured at some points of a story, which implied that the interpretation state is superposed or the measurement has a quantum nature. Qian and Dell [31] examined whether readers’ comprehension of sentences with late-closure ambiguity exhibits quantum-like properties. They asked readers about their incorrect (i.e., partial or locally coherent) understanding and their correct (i.e., globally coherent) understanding, and tested for the presence of order effects between these two questions. Their results showed no order effects, providing no support for a quantum-like comprehension state. Taken together with the findings of Fuyama [16,20], Qian and Dell [31]’s results suggest that when readers treat a text as having only “one correct answer,” their comprehension does not take on a quantum-like state. By contrast, for readings in which “there is no single correct answer” or “multiple interpretations are allowed,” such as metaphorical sentences or literary narratives, readers may be more likely to occupy a quantum-like state of understanding.

The existence of entanglement in a mind state has been tested using Bell-type inequalities such as CHSH inequalities [18,32]. One of the main difficulties in applying Bell-type inequalities to test entanglement in mental states is their lack of robustness against noise; several attempts have been made to address this limitation [33,34]. Related studies using linguistic corpora have been conducted in the domain of text comprehension. Surov et al. [17] modeled conceptual relations in text perception as an entanglement of a two-qubit state. They demonstrated a correlation between concurrence—an index of entanglement—and expert evaluations of the documents, providing empirical support for the hypothesis proposed in this study. We expect these findings to provide useful references.

### 4.2. Time Transition and the Algorithm

While reading, textual information continuously enters the reader’s comprehension state. Therefore, readers must construct a coherent state that integrates incoming information. Even when they believe that the constructed structure is valid, coherent, and represents the interpretation of the entire text, a single-twist sentence can dramatically alter their interpretation. Remarkably, readers seem able to grasp such twists and revise their entire interpretation of the story quickly; a detective story is a good example.

This article does not provide an explicit algorithm for implementing our hypothesis, the purification procedure, but indicates that these dynamics should serve as essential constraints when considering such an algorithm.

### 4.3. Adding the Dimension to Construct the Coherence

In the purification comprehension hypothesis, readers purify by adding an external system to enhance their overall coherence. In other words, from the perspective of von Neumann entropy, complexity is reduced by increasing the dimensionality of the system. This might seem strange when compared with common approaches, such as principal component analysis, which typically reduces dimensionality to uncover the structure. However, even in everyday visual perception, cognition often increases dimensionality to reconstruct a coherent structure of the world. For instance, we reconstruct a three-dimensional visual world from two-dimensional retinal input [35], and a Necker cube drawn on a flat surface is perceived as a three-dimensional object [36]. These examples indicate that embedding information in a higher-dimensional space can lead to a compressed informational representation. With respect to this example, increasing the dimensionality to reveal structures is not unusual in human cognition. From this perspective, our model may be extended beyond text comprehension to other modalities of aesthetic experience, such as appreciation of paintings or music, or even comprehension in general.

### 4.4. Appeal of Mixed States and Analyzing Text

The purification comprehension hypothesis may seem to regard the pure state as supreme and the mixed state as inferior. However, we emphasize that the mixed state directly evoked by the text has its own appeal in attracting the reader to purify the state. In this respect, one important future direction is to analyze the types of textual features that encourage purification and, conversely, the types that promote shallow reading, while recognizing that these effects may vary depending on the reader characteristics such as reading experience and reading style [37]. In addition, if multiple interpretations can be implemented in a semantic space model of texts [38], quantitative analysis of texts may also be fruitful. Corpus analyses related to quantum(-like) cognition can provide useful references [17,39].

### 4.5. Related Models and the Limitation of the Present Model: Hierarchical Structure and Distributed Model

The purification comprehension hypothesis models the entire coherence by employing quantum correlations. One limitation of this model is that it may not sufficiently capture hierarchical relations. By purification, the model produces an organized global structure that results in an information-compressed state in terms of von Neumann entropy without relying on the representation of propositions and rules for summarization as assumed by previous models featuring the macrostructure [7]. It remains unclear whether a hierarchical structure is required to explain the findings of previous macrostructure research [7] and suggestions from the humanities, such as the hermeneutic circle. Further consideration is required regarding the implication of “hierarchy” in text comprehension, as well as whether the present model itself can be extended to incorporate hierarchical structures.

Additionally, it is necessary to examine the relationship with distributed representations, which have become standard in recent language models [22,40]. Distributed representations are based on the distributional hypothesis according to which the meanings of words or sentences can be represented by their relationships with one another. They provide a representation of linguistic meaning that is distinct from language itself and, in this respect, may be considered analogous and/or competitive with the present model, which aims to represent meanings that are elicited from language but extend beyond it.

The present model primarily addresses reader interpretations, whereas distributed representation-based models have mainly been applied to text analysis or text generation tasks. The relationship between the two approaches is not immediately clear because of considerable differences in their representational formats. In future work, when the present model is examined in empirical studies, it may become possible to explore such connections, for example, by analyzing texts that evoke readers’ interpretation states using distributed representations.

## 5. Conclusions

In this article, we proposed a novel hypothesis, the purification comprehension hypothesis, which aims to explain how readers construct indescribable holistic comprehension by reading a text. This work contributed not only by providing a novel hypothesis but also by proposing a bridge between humanities and scientific studies approaches to the study of reading. According to this hypothesis, the reading process can be understood as purification, in which the reader finds and incorporates external components such as interpretations of other parts of the text or prior knowledge to purify their interpretation. Through this process, the dimensionality of the state increases, whereas von Neumann entropy decreases. This indicates potential connections with models that aim to minimize Shannon entropy and highlight the broader applicability of our hypothesis to comprehension across other modalities and media. Based on this hypothesis, we distinguish two types of reading, purification and measurement, and discuss their differences and relationships. Although our proposal remains purely theoretical, it provides a foundation for future empirical investigations. Such empirical studies will not only serve to test the hypothesis but also refine and improve it.

## Data Availability

This study is purely theoretical and does not involve the creation or analysis of datasets.
